# Age‐specific birth rates in women with epilepsy: a population‐based study

**DOI:** 10.1002/brb3.492

**Published:** 2016-06-14

**Authors:** Anette Huuse Farmen, Jacob Holter Grundt, Torbjörn Tomson, Karl O. Nakken, Jakob Nakling, Petter Mowinchel, Merete Øie, Morten I. Lossius

**Affiliations:** ^1^ Department of NeurologyInnlandet Hospital TrustLillehammerNorway; ^2^ National Center for EpilepsyOslo University HospitalNorway; ^3^ Department of PaediatricsOslo University Hospital RikshospitaletOsloNorway; ^4^ Department of Clinical NeuroscienceKarolinska InstituteStockholmSweden; ^5^ Department of GynaecologyInnlandet Hospital TrustLillehammerNorway; ^6^ Department of PaediatricsOslo University Hospital UllevålOsloNorway; ^7^ Institute of PsychologyUniversity of OsloNorway

**Keywords:** birth rates, fertility in epilepsy, pregnancy

## Abstract

**Objective:**

The aim of this study was to investigate birth rates and use of hormonal contraception in different age groups among women with epilepsy (WWE) in comparison to women without epilepsy.

**Materials and Methods:**

Demographic data and medical information on more than 25,000 pregnant women (40,000 births), representing 95% of all pregnancies in Oppland County, Norway, were registered in the Oppland Perinatal Database in the period 1989–2011. Data were analyzed with respect to epilepsy diagnoses, and 176 women with a validated epilepsy diagnosis (303 pregnancies) were identified. Age‐specific birth rates in these women were estimated and compared with age‐specific birth rates in women without epilepsy in the same county.

**Results:**

In WWE over 25 years of age, birth rates were significantly lower than in those of the same age group without epilepsy. In women below 20 years of age, birth rates were similar in those with and without epilepsy. The use of hormonal contraceptives prior to pregnancy was lower among WWE under 25 years than in the corresponding age group without epilepsy.

**Conclusions:**

Health professionals who counsel WWE who are of fertile age should be aware of the strongly reduced birth rates in WWE over 25 years of age, and the lower rates of use of contraceptives among young WWE.

## Introduction

Previous studies have reported that birth rates are lower among women with epilepsy (WWE) compared with those in the general population (Wallace et al. 1998; Artama et al. 2004). Hormonal changes and psychosocial problems have been discussed as potential causes (Wallace et al. 1998), but use of antiepileptic drugs (AEDs) may also be of significance. An Indian study that included women with validated epilepsy, reported AED polytherapy as a major risk factor for lack of conception, but this study did not compare birth rates to controls (Sukumaran et al. 2010). However, other studies indicate that controlling for comorbidities may be of greater significance regarding fertility rate than the epilepsy per se (Jalava and Sillanpaa 1997; Olafsson et al. 1998; Viinikainen et al. 2007). A Finnish study reported that reduced birth rates were related to active epilepsy during adulthood (active epilepsy defined as subjects taking AEDs), but birth rates did not otherwise differ from those in the reference group (Lofgren et al. 2009). The two largest population‐based studies reporting reduced birth rates in epilepsy relied on registry data, but without validation of the epilepsy diagnoses and without detailed clinical information such as epilepsy type and use of contraceptives (Wallace et al. 1998; Artama et al. 2004). One reported significantly lower birth rates in women aged 25–39 years with nonvalidated epilepsy and taking AEDs compared with birth rates in the general population (Wallace et al. 1998). The other study found 10% lower birth rates among WWE than women without epilepsy (Artama et al. 2004), but did not report any clear differences between different age groups, other than a general decline in fertility in the late 20s age group, as has also been reported in the general population (Dunson et al. 2002).

Given the partly conflicting results of previous studies and the lack of detailed clinical information, we aimed to study age‐dependent birth rates in a well‐described cohort of women with validated epilepsy.

The aim of our study was to compare mothers' age at first registered childbirth between women with and with‐out epilepsy, and to assess birth rates in different age groups among WWE. Additionally, we wanted to analyze the use of hormonal contraception prior to pregnancy.

## Materials and Methods

Information on pregnant women giving birth at either of the two obstetric departments in Oppland County (189,000 inhabitants), Norway, between 1989 and 2012, were prospectively registered in The Oppland Perinatal Database (OPD). This study includes data collected in the period between 1989 and 2011 on 25,203 women (data from the year 2012 were not processed and were mostly “raw data” at the beginning of the current project and therefore left out). The OPD includes information on approximately 95% of all pregnant women who have given birth within the county (main exceptions: births given in two small rural birth clinics, and women from one local community giving birth in a neighboring county obstetric department due to shorter distance). Demographic and medical information, including information on chronic medical conditions like epilepsy and use of hormonal contraception within 3 months before pregnancy, were systematically collected in conjunction with the routine ultrasound examination (week 17–19), at birth, and in the interim when necessary. Epilepsy was registered in a total of 346 pregnancies, either through pregnancy records or from direct questions about medical conditions at the time of ultrasound and at birth, and the data registered in OPD. After thorough validation (see below) 12.5% of pregnancies with registered epilepsy did not fulfill the ILAE‐criteria, thus 303 pregnancies by 176 women were included. Information about the study was sent by mail to all WWE registered in OPD, asking those not wishing to be included in the study to return the response letter. After passive consent from 166 of the 176 WWE, data from the 166 WWE were included in the dataset for further analysis. Only the first registered birth of each of the individual women was included in the analysis. The epilepsy diagnoses were validated by a neurologist (AHF), according to the ILAE criteria (Commission of Classification and Terminology of the International, 1989), by review of medical records and EEG reports. Women with epilepsy according to the ILAE criteria were included. Difficult‐to‐validate cases were discussed with an experienced epileptologist (MIL). Classification was carried out by three independent neurologists (KON, MIL, and AHF), and disagreements were resolved by discussions to consensus.

All women in OPD with a validated diagnosis of epilepsy from at least one point in their life prior to registration in the OPD were included in the study, since it is not unlikely that people with well‐controlled epilepsy and in whom AEDs have been withdrawn will also be affected by psychosocial implications of their epilepsy later in life (Camfield and Camfield 2007, 2014). Thus, all birth‐giving women with a history of epilepsy at some point in their lifetime were included in the analyses, irrespective of the presence or absence of ongoing treatment or time since last seizure.

It is possible that subjects with epilepsy in childhood only did not report the diagnoses, and thus missed inclusion. However, the frequency of epilepsy in the OPD was 0.6%, which is equivalent to the frequency in other birth registries (Viinikainen et al. 2007).

In order to estimate age‐specific birth rates in WWE in Oppland, we used the cumulative lifetime prevalence of epilepsy (12–13 per 1000 among 17 year olds) recorded in Akershus County, a similar area in the same part of Norway (Lossius et al. 2006). Total number of WWE in Oppland County was estimated using life time prevalence 12 per 1000 and population numbers in Oppland County (official numbers from Norway Statistics [www. SSB.no]), and then grouped in 5‐year age groups. The frequencies of births per 1000 women with and without epilepsy in each age group were then calculated based on the registered numbers of births from OPD. Age‐dependent birth rates in 5‐year age groups (from mothers aged 15–45 years at the time of birth giving) in women with and without epilepsy in OPD were then compared. Data regarding clinical variables and use of hormonal contraceptives were retrieved from the OPD.

The study was approved by the Regional Committee for Medical and Health Research Ethics, REC South East, Norway.

### Statistics

Possible group differences were tested using chi square test and independent *t*‐test for categorical and continuous variables, respectively. Poisson distributions with confidence intervals were used to analyze birth rates. A *P*‐value of 0.05 was used throughout. Microsoft Excel Version 2010 was used for calculations of age‐dependent birth frequencies within OPD. Data from OPD were plotted and processed in SPSS version 17, and statistical analyses were conducted using SPSS 17 (IBM SPSS Statistics, Intelytics Norge AS).

## Results

Among the WWE included in the study, 58% had focal epilepsy, whereas 29% had generalized epilepsy. In 13% the epilepsy was unclassifiable due to incomplete data. Interrater reliability in the classification of epilepsy was 78.4%. There were no significant differences in age‐dependent birth rates between the epilepsy subgroups. Fifty‐five percent of the women used AEDs throughout the pregnancy. Sample characteristics are shown in Table 1.

**Table 1:** Sample characteristics; age distribution, epilepsy classification, and antiepileptic drugs during pregnancy

Age (years)	15–19	20–24	25–29	30–34	35–39	40–44
Total number in each age group	13	54	57	29	12	1
Epilepsy classification: Generalized % (*n*)	38.5 (5)	29.6 (16)	21.1 (12)	31.0 (9)	31.8 (4)	100 (1)
Epilepsy classification: Focal % (*n*)	53.8 (7)	61.1 (33)	61.4 (35)	51.7 (15)	50.0 (6)	0
Epilepsy classification: Unclassified % (*n*)	7.7 (1)	9.3 (5)	17.5 (10)	17.2 (5)	18.2 (2)	0

Age (years)	15–19	20–24	25–29	30–34	35–39	40–44
Antiepileptic drugs during pregnancy % (*n*)	53.8 (7)	57.4 (31)	54.5 (31)	41.4 (12)	75 (9)	100 (1)
Valproate % (*n*)	15.4 (2)	7.4 (4)	12.3 (7)	10.3 (3)	13.6 (2)	0
Carbamazepine % (*n*)	23.1 (3)	29.6 (16)	15.8 (9)	14.0 (4)	36.4 (4)	0
Lamotrigine % (*n*)	7.7 (1)	7.4 (4)	7.0 (4)	6.9 (2)	13.6 (2)	100 (1)
Polytherapy % (*n*)	7.7 (1)	9.3 (5)	8.8 (5)	10.3 (3)	0	0
Other antiepileptic drugs^a^, *n*	0	2	6	0	1	0

a Levetiracetam, Topiramat, Phenytoin, Clobazam.

**Figure 1:**
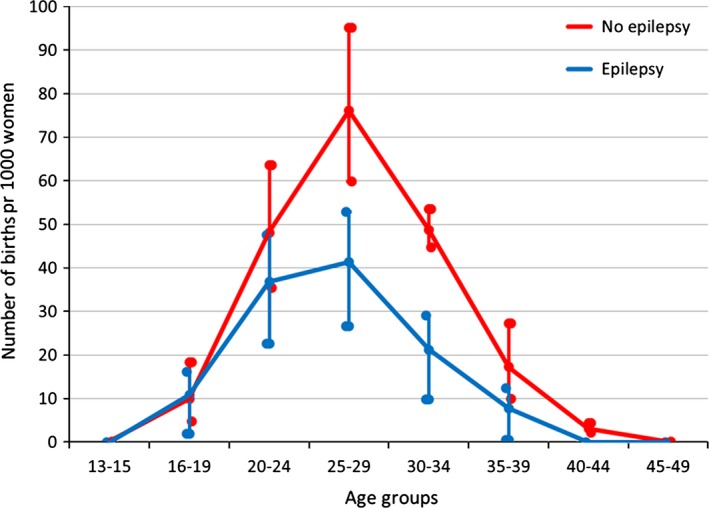
Number of births per 1000 women in Oppland Perinatal Database during 1989–2011.

Between the ages 25 and 35 years, birth rates among WWE were significantly lower (*P* < 0.001) than in the population of women without epilepsy in OPD (Fig. 1). Birth rates were still lower in WWE older than 35 years, but not significantly. Before the age of 20 years the birth rates in women with and without epilepsy in OPD were similar. Compared with women without epilepsy in OPD, WWE were significantly younger (mean 26.4 years vs. 27.9 years, *P* < 0.001, CI 2.29–0.71).

Women with epilepsy used hormonal contraceptives (pills, implants, or intrauterine devices) less often than those without epilepsy, except in the oldest age group (between 35 and 44 years). The differences were most evident, and statistically significant, in the youngest age groups (15–24 years), where only 17% used hormonal contraceptives compared with 35% of women without epilepsy in OPD (*P* = 0.01) (Table 2).

**Table 2:** Use of hormonal contraception in Oppland Perinatal Database

Age, years	15–24	25–34	35–44	Total
Epilepsy (*n* = 129)	17.3% (9/52)	22.6% (15/67)	12.5% (1/10)	19%
Controls (*n* = 20 897)	34.5% (1535/4449)	26.4% (3580/13562)	12.5% (361/2884)	27%
*P*	0.01	0.50	0.77	0.07

## Discussion

The most important finding in this study is that birth rates among WWE in OPD were reduced in all age groups older than 20 years compared with the women without epilepsy, and this difference in birth rate was significant between 25 and 35 years. Before the age of 20 years there was no difference in birth rates. Our findings support earlier studies showing that epilepsy may be a predisposing factor for not having children, and that WWE have significantly reduced birth rates after 25 years of age (Wallace et al. 1998). The results thus corroborate the findings from earlier population‐based studies (Wallace et al. 1998; Artama et al. 2004). In addition, this study is based a material of women with a validated epilepsy diagnosis, including detailed information on AED use and the use of contraceptives. Various factors might be postulated to explain the reduced birth rate in WWE older than 20 years. The epilepsy etiology, the epilepsy duration, and type of AED used and duration of drug treatment are all likely to affect hormonal cycles and the metabolism of sex hormones, and thus sexual function, including libido (Schupf and Ottman 1996; Stoffel‐Wagner et al. 1998; Klein et al. 2001; Harden and Pennell 2013). Premature menopause in WWE has also been reported (Klein et al. 2001). Psychosocial factors, such as difficulties in finding a partner, low self‐esteem, stigmatization, and social isolation may also play a substantial role in many cases. Comorbidities occur frequently in people with epilepsy, particularly anxiety and depression (Lossius et al. 2006; Alfstad et al. 2011), and could be associated. Furthermore, fear of fetal malformations as a consequence of AED use during pregnancy, fear of seizures during pregnancy, and fear of the risk of epilepsy in the child may also be potentially contributing factors.

Before the age of 20 years there was no difference in birth rates between WWE and women without epilepsy in OPD, and in the 20–24 years age group there was a smaller relative difference than in older age groups. These results indicate that WWE who become pregnant, do so earlier in life compared with the general population, but which specific mechanisms are of relevance are not clear. The apparently normal birth rate in younger ages may be due to biological factors, such as shorter duration of epilepsy at younger age, or behavioral factors, like less use of hormonal contraception. Bio‐pharmaceutical factors such as failure of contraceptives due to interactions with AEDs would apply for older women as well. There is no reason to believe that comorbidity is less common among young people with epilepsy (Lossius et al. 2006; Alfstad et al. 2011). A Norwegian study suggested that young adults with epilepsy are more prone to risk‐taking behavior, discussing whether epilepsy may be an independent risk factor for early sexual debut (Alfstad et al. 2011). Whether risk‐taking behavior also may explain the reduced use of contraceptives is unknown, but in our data the difference in hormonal contraception usage was most evident in the youngest age group. Additionally, the birth rate among WWE aged between 15 and 20 years might be explained by fewer pregnancy‐related fears or the tendency toward increased risk‐taking behavior due to immature executive functions in adolescence in general (Sommerville et al. 2010). Thus, fears of epilepsy‐related consequences may be less prominent in younger WWE, while these concerns may increase with age as frontal, executive functions mature (Sommerville et al. 2010).

The strengths of this study are the detailed clinical data of the epilepsy population included in the study, validation of the diagnoses, and reliable information about AED treatment and recorded information on hormonal contraception. The limitations of the study include the relatively low numbers of WWE in each age group (Table 1), and the uncertainties associated with cumulative incidence of epilepsy as increasing lifetime prevalence with increasing age was not taken into account. However, if the increasing prevalence with age had been included, then the difference between the WWE and the controls would have been even greater. We estimated birth rates based upon registry data from OPD and the lifetime prevalence value (active and earlier epilepsy) 12 per 1000 from a Norwegian county geographically and demographically similar to ours (Lossius et al. 2006). A study from Oppland County in 2007 reported an age‐specific prevalence of active epilepsy to be 9.7 per 1000 among those born in 1970 (35 years at the time of study), and 7.5 per 1000 in those born in 1960 (Svendsen et al. 2007). Wallace et al. (1998) reported an epilepsy prevalence of 5.2–5.4 per 1000 in an age group of 15–44 years. Both these studies were based on active epilepsy, in contrast to the OPD data that focuses on cumulative incidence. A recent Irish study found self‐reported lifetime prevalence of 10 per 1000 among adults (Linehan et al. 2009). Based on these reports, the estimated cumulative incidence of 12 per 1000 appears reasonable.

## Conclusions

Our study assessed birth rates and the use of contraceptives in different age groups in a well‐described, classified, and validated cohort of WWE in Norway. The results indicate that WWE who become pregnant, do so earlier in their life than women without epilepsy, and that the probability of WWE having their first child after the age of 25 years is significantly reduced. The lower use of contraceptives among young WWE calls for special attention in counseling.

## Conflict of Interest

T. Tomson has received speakers honoraria for his institution from Eisai, UCB, and Actavis, honoraria for his institution for advisory boards from UCB and Eisai, and received research support from Stockholm County Council, CURE, GSK, UCB, Eisai, Bial, and Novartis. M.I. Lossius has been involved in expert panels for EISAI and given lectures for UCB and ESAI. The remaining authors have no conflicts of interest.
